# Comparative evaluation of three polymerase chain reaction primer sets for accurate molecular detection of *Trypanosoma lewisi* in wild rodents in Indonesia

**DOI:** 10.14202/vetworld.2025.2395-2405

**Published:** 2025-08-21

**Authors:** Aditya Yudhana, Gusti Ayu Illiyin Putri Santosa, April Hari Wardhana, Frenky Laksana Putra, Ryanka Edila, Dyah Haryuningtyas Sawitri, Ratih Novita Praja, Muhammad Aqil Kurnianto, Aldi Gusnizar Rizaldy Tanjung, Marc Desquesnes, Makoto Matsubayashi

**Affiliations:** 1 Department of Health and Life Sciences, Veterinary Medicine Study Program, Faculty of Health, Medicine, and Life Sciences, Universitas Airlangga, Banyuwangi 68425, East Java, Indonesia; 2 Research Group for Animal Biomedical and Conservation, Universitas Airlangga, Banyuwangi 68425, East Java, Indonesia; 3 Department of Veterinary Science, Graduate School of Veterinary Science, Osaka Metropolitan University, 1-58 Rinku Orai Kita, Izumisano, Osaka 598-8531, Japan; 4 Research Center for Veterinary Science, Organization for Health, National Research and Innovation Agency, Cibinong 16911, West Java, Indonesia; 5 Master Program of Veterinary Disease and Veterinary Public Health, Faculty of Veterinary Medicine, Universitas Airlangga, Surabaya 60115, East Java, Indonesia; 6 Doctoral Program of Veterinary Science, Faculty of Veterinary Medicine, Universitas Airlangga, Surabaya 60115, East Java, Indonesia; 7 Université de Montpellier, CIRAD, IRD, Intertryp, Montpellier, France; 8 Agricultural Research for the Sustainable Development of Tropical and Mediterranean Regions, Joint Research Unit on Trypanosomatids, 31076 Toulouse, France; 9 Department of Veterinary Science National Veterinary School of Toulouse (ENVT), 23 Chemin des Capelles, 31000, Toulouse, France

**Keywords:** diagnostic validation, flea-transmitted protozoa, molecular diagnostics, neglected disease, polymerase chain reaction primers, public health, rodent-borne zoonosis, Southeast Asia, *Trypanosoma lewisi*

## Abstract

**Background and Aim:**

*Trypanosoma lewisi* is a flea-transmitted protozoan parasite commonly infecting rodents and posing zoonotic risks. Conventional diagnostics such as blood smear and serology often fail in low parasitemia conditions. Molecular diagnostics using polymerase chain reaction (PCR) offer improved sensitivity and specificity, but the optimal primer set for field detection remains unclear. This study aimed to compare the diagnostic performance of three published PCR primer sets–TC121/TC122, CATLew F/CATLew R, and LEW1S/LEW1R–for the detection of *T. lewisi* in wild *Rattus* spp. in Indonesia and determine the most reliable tool for field application.

**Materials and Methods:**

One hundred rat blood samples obtained from the Badan Riset dan Inovasi Nasional (BRIN), Research Center for Veterinary Science, Bogor, West Java Province, Indonesia were analyzed through PCR using the three primer sets under optimized thermal cycling conditions. DNA amplification products were visualized using agarose gel electrophoresis. Diagnostic performance was evaluated based on sensitivity and specificity calculations using microscopy as the reference standard.

**Results:**

The LEW1S/LEW1R primer set demonstrated the highest diagnostic accuracy, detecting *T. lewisi* in 30 samples with 100% sensitivity and 97.22% specificity. CATLew F/CATLew R detected 29 positives with 96.43% sensitivity and 97.22% specificity, whereas TC121/TC122 detected 21 positives, yielding 67.86% sensitivity and 97.22% specificity. Only the LEW1S/LEW1R primer set consistently produced single, distinct amplicons with no non-specific bands.

**Conclusion:**

LEW1S/LEW1R is the most sensitive and diagnostically reliable primer set for PCR-based detection of *T. lewisi*, particularly suitable for low-resource settings where accurate and early detection is crucial. Its implementation in surveillance programs can strengthen zoonotic disease monitoring and guide timely interventions. Future studies should validate these findings in mixed-infection contexts and explore their application in human and non-rodent hosts.

## INTRODUCTION

Rodents are among the most adaptable mam-malian species, inhabiting a wide range of ecological niches and environmental conditions [1]. Despite their behavioral, morphological, and ecological diversity, they share a unique characteristic–ever-growing incisors that require constant gnawing for maintenance [2, 3]. Rodents serve as reservoirs for a multitude of micro-organisms, including bacteria, viruses, protozoa, hel-minths, fungi, and ectoparasites, many of which are zoonotic and pose significant public health risks [4, 5]. Their ecological versatility, combined with their capacity to harbor and transmit pathogens, positions rodents as key contributors to both ecosystem balance and disease transmission dynamics [6, 7].

*Trypanosoma* spp. are protozoan parasites respo-nsible for trypanosomosis in both humans and ani-mals. These parasites are typically transmitted through hematophagous arthropod vectors, such as tsetse flies, horseflies (*Tabanus*), reduviid bugs, mites, and fleas [8, 9]. Notably, several flea genera–*Xenopsylla*, *Ctenophthalmus*, *Nosopsyllus*, and *Dinopsylla*–have been identified as competent vectors worldwide [10–12]. Among the *Trypanosoma* species associated with rod-ents, *Trypanosoma* *lewisi*, *Trypanosoma* *evansi*, and *T. lewisi*-like organisms have been documented across multiple continents, including Europe, Asia, Australia, the Americas, and Africa, highlighting their extensive distribution and zoonotic potential [13–15].

*T. lewisi*, a non-pathogenic member of the sub-genus *Herpetosoma*, primarily infects rodent hosts and is transmitted through flea vectors [16]. Its global dissemination is closely associated with the spread of commensal rodents, a process facilitated by human travel and commercial activity [17]. This underscores the influence of anthropogenic factors on the transmission dynamics of *T. lewisi*, emphasizing the intricate inter-section between ecology and public health [18].

Reports of *T. lewisi* prevalence in rodent popu-lations vary geographically. For instance, infection rates of 54% in *Rattus* *rattus* and 4% in *Rattus* *norvegicus* have been observed in Italy [19]. In Southeast Asia, prevalence rates include 1.5% in rats from traditional markets in Malaysia [20], and 16.7%, 9.5%, and 12.4% in Thailand, Cambodia, and Myanmar, respectively [21]. A study in Vietnam further reported a striking 62.5% prevalence among rodents captured in high-traffic environments such as hospitals and marketplaces [22]. These data illustrate the remarkable adaptability of *T. lewisi*, especially in densely populated urban settings.

Moreover, atypical human infections involving *T. lewisi*, *T. lewisi*-like organisms, and *T. evansi* have been recorded in countries such as Malaysia, Sri Lanka, India, and Thailand [23, 24], heightening concerns over their zoonotic implications. To date, 44 *Trypanosoma* species have been identified in 144 rodent host species, predominantly within the Stercoraria section [25], sugg-esting a wide host range and potential for cross-species transmission [26].

In Indonesia, however, data on *T. lewisi* remain sparse, with existing studies limited to a few regions, including Malang, South Sulawesi, Banjarnegara, Sura-baya, and Banyuwangi [27]. Banyuwangi, located along the South Eastern coast of Java, is characterized by dense human populations, impoverished communities, and substandard sanitation conditions [28, 29], making it a critical area for examining *T. lewisi* ecology. Urbanization and land-use changes in this region may further modify rodent-vector-human interactions, increasing the risk of parasite spillover and altering disease transmission patterns [30, 31]. Understanding these ecological relationships is essential for designing effective control strategies and assessing emerging zoonotic threats.

A variety of diagnostic approaches are available for detecting *T. lewisi* in rodents, each with distinct strengths and limitations. Among these, molecular techniques are increasingly preferred due to their superior specificity and diagnostic accuracy. Commonly employed primer sets include TRYP1, which targets the internal transcribed spacer 1 (ITS1) region of ribosomal DNA across multiple *Trypanosoma* species; TBR, which amplifies satellite DNA specific to the *Trypanozoon* sub-genus; and LEW1, designed to detect the ITS1 region specifically in *T. lewisi* [22, 32, 33].

Although *T. lewisi* has been detected in rodent populations across various parts of Asia, Africa, and Europe, its prevalence and transmission dynamics in Indonesia remain significantly underexplored. Exis-ting studies in the country have been limited to a handful of regions, including Malang, South Sulawesi, Banjarnegara, Surabaya, and Banyuwangi, and these investigations have largely relied on conventional diagnostics such as blood smear microscopy. While microscopy remains a cost-effective screening tool, its sensitivity is considerably diminished in cases of low parasitemia, which are common in chronic or subclinical *T. lewisi* infections. Furthermore, although molecular diagnostics–especially polymerase chain reaction (PCR)–have emerged as highly accurate tools for protozoan detection, there is a lack of comparative data on the performance of different primer sets specifically targeting *T. lewisi*. Current literature often reports isolated findings using individual primer sets, without standardized comparisons to determine their relative sensitivity, specificity, and field applicability. This lack of comparative validation limits the ability to choose the most reliable and cost-effective diagnostic approach for field surveillance and zoonotic risk assessment, especially in resource-limited settings such as many regions in Indonesia. In addition, the zoonotic potential of *T. lewisi*–highlighted by emerging reports of atypical human infections–underscores the urgent need to improve early detection strategies for both veterinary and public health monitoring.

In response to these gaps, the present study aimed to perform the first comparative evaluation of three published PCR primer sets–TC121/TC122, CATLew F/CATLew R, and LEW1S/LEW1R–for the molecular detection of *T. lewisi* in wild Rattus spp. in Indonesia. Specifically, this study sought to: (1) conduct a field-based molecular survey of *T. lewisi* in urban rodent populations from Banyuwangi, a coastal region with high zoonotic risk due to dense human settlement and poor sanitation; (2) compare the sensitivity and specificity of the three primer sets under standardized laboratory conditions using PCR amplification and electrophoretic analysis; and (3) identify the most diagnostically effective primer set for use in future surveillance pro-grams. By evaluating these primers side-by-side, this study provides essential baseline data to inform the selection of optimal molecular diagnostics for *T. lewisi*, with direct implications for improving zoonotic disease monitoring, reducing diagnostic errors, and guiding control efforts in Indonesia and similar ecological set-tings in Southeast Asia.

## MATERIALS AND METHODS

### Ethical approval

This study was conducted in accordance with ethical standards. Ethical approval was granted by the Ethical Clearance Committee of the Faculty of Veterinary Medicine, Universitas Gadjah Mada, under certificate number 055/EC-FKH/Eks./2023.

### Study period and location

The study was conducted from May to December 2024. The samples were analyzed at the Molecular Labo-ratory located in Bogor, West Java Province, Indonesia.

### Sample collection and preliminary screening

A total of 100 blood samples from wild rats (*Rattus* spp.) were obtained from the Badan Riset dan Inovasi Nasional (BRIN), Research Center for Veterinary Science, Bogor, West Java Province, Indonesia. These stock samples had been collected in 2023 from slum areas in Banyuwangi, East Java Province, Indonesia–an environment characterized by high rodent density and suboptimal sanitation. Each sample was initially screened for *T. lewisi* using conventional blood smear microscopy by trained personnel at BRIN. After microscopic screening, the blood samples were preserved in crookes tubes and stored at −20°C for subsequent molecular analysis.

### Genomic DNA extraction

Genomic DNA was extracted from 300 μL of each whole blood sample using the Genomic DNA Mini Kit (Geneaid, Taiwan), following the manufacturer’s protocol. The resulting DNA extracts were transferred into 1.5 mL labeled microcentrifuge tubes (Eppendorf, Germany) and storedat −20°C until further use for PCR amplification [33].

### PCR amplification

Three published primer sets–TC121/TC122 [34], CATLew F/CATLew R [35], and LEW1S/LEW1R [32]–were employed to detect *T. lewisi* DNA. The expected ampli-con sizes were 700 bp, 253 bp, and 220 bp, respectively. PCR was carried out using a Biometra Tone thermal cycler.

#### The primer sequences used were as follows

- TC121: 5’-AAA TAA TGT ACG GG(T/G) GAG ATG CAT GA-3’
- TC122: 5’-GGT TCG ATT GGG GTT GGT GTA ATA TA-3’
- CATLew F: 5’-ACA GTG GTA CCT CGC CGG CCA TAA-3’
- CATLew R: 5’-CTG CGG CAG GTC AAC GTA GTC CTT-3’
- LEW1S: 5’-ACC ACC ACA CGC TCT CTT CT-3’
- LEW1R: 5’-TGT ATG TGC GTG CTT GTT CA-3’

Each 25 μL PCR reaction contained MyTaq^™^ HS Mix (Bioline, UK), the appropriate primer pairs, DNA template, and nuclease-free water. Positive and negative controls were included in all reaction sets to monitor for contamination and confirm amplification of the target DNA fragment. Thermal cycling conditions specific to each primer set are provided in Table 1 [6, 32, 34].

**Table 1:** PCR conditions of the three primers tested in this study.

PCR condition	TC 121/TC122	CATLew F/R	LEW1S/LEW1R
Pre denaturation	98°C/1 min	94°C/3 min	94°C/2 min
Denaturation	98°C/30 s	94°C/30 s	94°C/30 s
Annealing	64°C/30 s	63°C/30 s	56°C/30 s
Elognation I	72°C/1 min	72°C/1 min	72°C/1 min
Elognation II	72°C/3 min	72°C/3 min	72°C/3 min
Final	4°C/∞	4°C/∞	4°C/∞
Comments	35 cycles	35 cycles	35 cycles
Reference	[34]	[6]	[32]

PCR=Polymerase chain reaction

### Gel electrophoresis and visualization

PCR products were analyzed by agarose gel elect-rophoresis. For each reaction, 5 μL of amplified DNA was loaded onto a 1.5% Tris, Acetic acid, and EDTA (TAE) (Thermo Scientific, USA) agarose gel alongside a 3,000 bp DNA ladder. The gel was stained using Fluoro Safe DNA stain (1^st^ Base) and electrophoresed at 100 volts for 30 min. DNA bands were visualized under ultraviolet light using a GelDoc transilluminator (Cleaver Scientific, USA) [33].

### Statistical analysis

Results were recorded and tabulated using Micro-soft Excel 2013. Diagnostic test performance was evaluated by calculating sensitivity and specificity using the following formulas:

- Sensitivity = (A/[A + C]) × 100%
- Specificity = (D/[B + D]) × 100%

Where, A = True positives, B = False positives, C = False negatives, and D = True negatives. The 95% confidence intervals (CIs) were calculated for each parameter to ensure statistical accuracy (Table 2) [35].

**Table 2:** Screening test and disease status [35].

Screening test	Result	Disease status		

		Present	Absent	Total
	Positive	A	B	A + B
	Negative	C	D	C + D
	Amount	A + C	B + D	A + B + C + D

A: Number of true positives (detected positive for *T. lewisi* based on gold standard blood test and PCR). B: Number of false negatives (detected positive for *T. lewisi* based on gold standard blood test, detected negative for *T. lewisi* based on PCR). C: Number of false positives (detected negative for *T. lewisi* based on gold standard blood test, detected positive for *T. lewisi* based on PCR). D: Number of true negatives (detected negative for *T. lewisi* based on gold standard blood test and PCR). PCR=Polymerase chain reaction, *T. lewisi=Trypanosoma lewisi*

## RESULTS

### PCR detection using TC121/TC122 primer set

**Figure 1:**
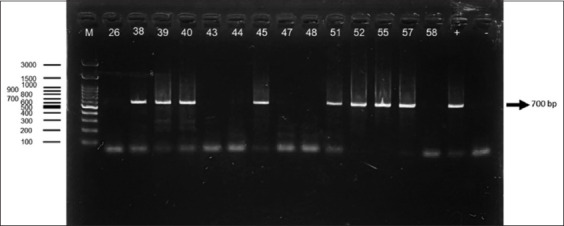
The electrophoresis results of rat blood samples using TC 121/TC 122 primers [34]. The band at approximately 700 bp (indicated by the arrow) corresponds to the expected amplicon size for *Trypanosoma lewisi*. (m): Marker, (26, 38, 39, 40, 43, 44, 45, 47, 48, 51, 52, 55, 57, 58): Indicates sample number, (+): Positive control, (−): Negative control.

Out of 100 wild rat blood samples analyzed by PCR, the TC121/TC122 primer set detected *T. lewisi* in 21 samples. The resulting amplicons corresponded to the expected fragment size of 700 base pairs, confirming successful amplification of the target seq-uence [34]. However, this primer set demonstrated the lowest detection rate among the three evaluated. The electrophoresis results using TC121/TC122 are pre-sented in Figure 1 [34].

### PCR detection using CATLew F/CATLew R primer set

**Figure 2:**
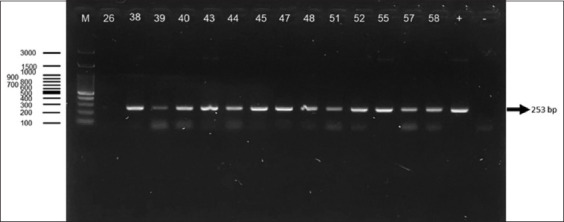
The electrophoresis results of rat blood samples using CATLew F/CATLew R [6]. The band at approximately 230 bp (indicated by the arrow) corresponds to the expected amplicon size for Trypanosoma lewisi. (m): Marker, (26, 38, 39, 40, 43, 44, 45, 47, 48, 51, 52, 55, 57, 58): Indicates sample number, (+): Positive control, (−): Negative control.

The CATLew F/CATLew R primer set showed impr-oved performance over TC121/TC122, detecting *T. lewisi* in 29 of the 100 samples. A distinct 253-bp band was observed in all positive samples, indicating consistent amplification and high primer specificity. These results suggest that the CATLew F/CATLew R set is a more reli-able tool for *T. lewisi* detection. The corresponding gel image is shown in Figure 2 [6].

### PCR detection using LEW1S/LEW1R primer set

**Figure 3:**
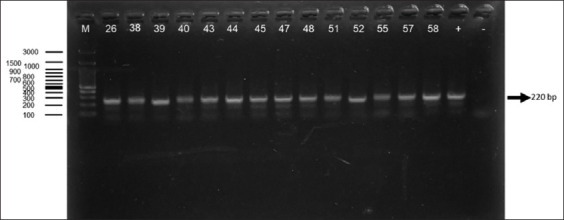
The electrophoresis results of rat blood samples using LEW1S/LEW1R [32]. The band at approximately 220 bp (indicated by the arrow) corresponds to the expected amplicon size for *Trypanosoma lewisi*. (m): Marker, (26, 38, 39, 40, 43, 44, 45, 47, 48, 51, 52, 55, 57, 58): Indicates sample number, (+): Positive control, (−): Negative control.

Among the three primer sets, LEW1S/LEW1R exhi-bited the highest diagnostic performance, identifying *T. lewisi* DNA in 30 samples. All positive samples produced a single, clear band at 220 base pairs, con-firming both high sensitivity and specificity [32]. This primer set consistently outperformed the others in both detection rate and band clarity. The PCR results for LEW1S/LEW1R are shown in Figure 3 [32].

### Comparative detection rates and diagnostic accuracy

Table 3 presents a summary of the detection rates for all three primer sets. The number of positive detections varied, indicating differences in diagnostic sensitivity and specificity. These were quantitatively assessed using standard diagnostic test formulas [35].

**Table 3:** Summary of *Trypanosoma lewisi* detection in wild rat blood samples using blood smear examination and three different PCR primer sets.

No.	Primer	Positive	Negative	Total	95% CI (%)
1	Blood smear*	28	72	100	19.9–37.8
2	TC121/TC122	21	79	100	13.9–30.3
3	CATLew F/CATLew R	29	71	100	20.8–38.8
4	LEW1S/LEW1R	30	70	100	21.7–39.7

The table shows the number of positive and negative samples, total samples tested, and corresponding 95% CI for each method.

* : Blood tests are considered the gold standard for Trypanosomosis testing. A blood test was conducted by BRIN researchers. PCR=Polymerase chain reaction, CI=Confidence interval

- LEW1S/LEW1R achieved the highest diagnostic accuracy with 100% sensitivity and 97.22% spe-cificity, indicating its ability to detect all true posi-tives with minimal false positives.
- CATLew F/CATLew R also showed strong perfor-mance, with a sensitivity of 96.43% and specificity of 97.22%, making it a viable alternative when LEW1S/LEW1R is unavailable.
- TC121/TC122, despite its acceptable specificity (97.22%), showed a markedly lower sensitivity of 67.86%, suggesting a greater likelihood of false-negative results and limited diagnostic utility in field surveillance.

### Conclusion on primer performance

Based on this comparative evaluation, the LEW1S/LEW1R primer set stands out as the most diag-nostically effective for PCR-based detection of *T. lewisi* in wild rat populations. Its high sensitivity and specificity, along with clear electrophoretic results, position it as the preferred molecular tool for accurate surveillance and diagnosis. Table 4 provides a detailed comparison of the sensitivity and specificity values of all three primer sets.

**Table 4:** Sensitivity and specificity of three PCR primer sets for detecting *Trypanosoma lewisi* in wild rat blood samples.

No.	Primer types	Size (bp)	Sensitivity (%)	95% CI (%)	Specificity (%)	95% CI (%)
1	TC 121/TC 122	700	67.86	49.3–82.1	97.22	89.4–99.7
2	CATLew F/CATLew R	253	96.43	81.7–99.9	97.22	89.4–99.7
3	LEW1S/LEW1R	220	100	87.9–100	97.22	89.4–99.7

The table includes the expected PCR product size (base pairs), sensitivity, and specificity percentages, along with their respective 95% CI. PCR=Polymerase chain reaction, CI=Confidence intervals

## DISCUSSION

### Advantages of PCR over conventional diagnostic methods

This study employed a molecular PCR-based approach to detect *T. lewisi* in wild rats (*Rattus* spp.), confirming its reliability and diagnostic efficiency. PCR provides significant advantages over traditional tools such as microscopy and serology. Unlike microscopy, which requires high parasitemia levels and is prone to subjective interpretation, PCR offers higher sensitivity and specificity, enabling detection even at low para-sitemia levels [36–38]. Moreover, PCR results are obtainable within hours, compared to the extended processing time needed for culture-based methods or microscopic examination [37]. Its ability to target specific DNA sequences reduces the likelihood of false positives, which is a common limitation of serological assays due to cross-reactivity [39].

PCR can also be multiplexed to detect vari-ous pathogens simultaneously, which is especially advantageous in endemic regions where co-infections with different *Trypanosoma* species are likely [40].

### Overall primer performance and diagnostic metrics

All three primer sets evaluated–TC121/TC122, CATLew F/CATLew R, and LEW1S/LEW1R–were capable of detecting *T. lewisi*, though with varying degrees of sensitivity and specificity. Sensitivity and specificity are critical for evaluating diagnostic performance. A test with high sensitivity detects nearly all true positives, while high specificity ensures few false positives [41, 42]. Among the primers tested, LEW1S/LEW1R achieved the highest diagnostic accuracy, with 100% sensitivity and 97.22% specificity, making it the most reliable detection tool for *T. lewisi* in this study. CATLew F/CATLew R also demonstrated strong diagnostic validity (sensitivity: 96.43%; specificity: 97.22%) and could serve as an effective alternative. In contrast, TC121/TC122 showed the lowest sensitivity (67.86%) despite maintaining similar specificity (97.22%), indicating a greater likeli-hood of false-negative results.

### Performance of the TC121/TC122 primer set

The TC121/TC122 primer set successfully amplified a 700 bp fragment and detected *T. lewisi* in 21 out of 100 samples. However, its relatively low sensitivity limits its diagnostic reliability. Originally designed for detecting *T. cruzi* via kinetoplast DNA (kDNA), the TC121/TC122 primers are not *T. lewisi*-specific [43]. Because both parasites belong to the Stercoraria section of *Trypanosoma*, they share homologous kDNA regions, which may explain the cross-reactivity and reduced specificity observed [24, 44]. Furthermore, this primer set can also amplify DNA from *T. rangeli*, a member of the Salivaria group [45], thus increasing the risk of non-specific amplification. These limitations make the TC121/TC122 set less suitable for reliable detection of *T. lewisi*, particularly in field surveillance studies.

### CATLew F/CATLew R primer set: Strengths and limitations

The CATLew F/CATLew R primers amplified a 253 bp fragment of the *Cathepsin L* (*CATL*) gene in 29 samples, demonstrating higher sensitivity (96.43%) than TC121/TC122. While the *CATL* gene offers good specificity and has been used to detect a range of *Trypanosoma* species, including *T. vivax*, *T. rangeli*, *T. cruzi*, *T. theileri*, and *T. congolense* [35, 46–54], the primer set in this study occasionally amplified multiple bands during electrophoresis. These non-specific bands suggest that the primers may not be highly specific under conditions of low parasitemia. Although CATLew F/CATLew R remains a valuable tool, especially in moderate to high parasitemia settings, its performance is limited when used as a confirmatory diagnostic method.

### LEW1S/LEW1R primer set: Superior diagnostic utility

The LEW1S/LEW1R primers outperformed all others, detecting *T. lewisi* in 30 samples by amplifying a clear 220 bp band. No non-specific amplification was observed, demonstrating excellent specificity and clarity in gel resolution [21, 32, 33]. These primers are designed from the ITS1 region, modified specifically for *T. lewisi* based on primers originally used for *T. evansi*. Their high sensitivity enables detection at picogram DNA concentrations (0.055–0.55 pg), equivalent to as few as 1–10 organisms per reaction [32]. These qualities make the LEW1S/LEW1R primers especially suitable for detecting low-level infections in both laboratory and field settings, including atypical human cases and wildlife surveillance [18, 41].

### Critical considerations in primer selection for PCR

Primer performance in PCR is influenced by several factors:

- Target DNA nature: Primers targeting repetitive sequences often exhibit better sensitivity than those targeting single-copy genes.
- Primer specificity: Excessive specificity may lead to false negatives if closely related species are not amplified [41].
- Sample parasitemia: Low parasitemia (<1–10 parasites/mL) is common in chronic infections and carriers, increasing false-negative risk.
- Contamination: Carryover contamination from positive samples can cause false positives, especially in nested PCR setups [22].
- PCR inhibitors and DNA overload: Overloading the reaction can suppress amplification due to contaminants.

To minimize these risks, selecting primers like LEW1S/LEW1R with validated high sensitivity and spe-cificity is essential, especially when screening for atypical trypanosomosis in both humans and animals [55–59].

### Limitations of microscopy and the value of molecular confirmation

Although blood smear microscopy remains a wid-ely accepted method, it is limited by poor sensitivity in low parasitemia cases, often yielding false negatives. Consequently, PCR should be employed as a confirmatory tool to avoid misdiagnosis. In this study, LEW1S/LEW1R demonstrated superior diagnostic reliability, reinforcing the need for molecular confirmation in epidemiological surveys.

### Technical aspects affecting PCR accuracy

Several technical elements must be optimized for accurate PCR detection:

- Primer design: GC content should range from 40% to 60%, and melting temperatures (Tm) for forward and reverse primers should be closely matched (<5°C difference) [60, 61].
- DNA storage: Low-temperature storage and proper buffer use prevent degradation [62].
- Extraction quality: Inefficient extraction or protein contamination can inhibit amplification [63].

Proper optimization of these parameters is crucial for enhancing diagnostic performance in field and laboratory applications.

### Universal primers versus specific primers for *T. lewisi*

Universal primers such as TRYP1S/R have been used to detect multiple *Trypanosoma* spp., including *T. lewisi* and *T. evansi* [64, 65]. However, they often amplify unintended sequences, leading to non-specific bands. In contrast, LEW1S/LEW1R offers superior speci-ficity and sensitivity, making it the preferred choice for targeted surveillance.

### Implications for disease surveillance and public health

The LEW1S/LEW1R primer set holds substantial potential for improving surveillance of trypanosomosis in low-resource and endemic settings. Its high reliability supports early diagnosis, reduces the need for retesting, and minimizes costs–essential features for public health interventions. This study is the first to comprehensively compare the sensitivity and specificity of three widely used primer sets for *T. lewisi* detection in wild rodents from Indonesia, offering critical insights applicable across Southeast Asia. These findings address a major diagnostic gap and contribute to improve molecular detection strategies for zoonotic trypanosomosis.

## CONCLUSION

This study presents the first comparative evalu-ation of three widely used PCR primer sets–TC121/TC122, CATLew F/CATLew R, and LEW1S/LEW1R–for the molecular detection of *T. lewisi* in wild *Rattus* spp. in Indonesia. Among the 100 wild rat blood samples analyzed, the LEW1S/LEW1R primer set demonstrated the highest diagnostic performance, detecting 30 positive samples with 100% sensitivity and 97.22% specificity. CATLew F/CATLew R also performed reli-ably, detecting 29 positives with 96.43% sensitivity and 97.22% specificity, while TC121/TC122 showed significantly lower sensitivity (67.86%) with the same specificity (97.22%), reflecting a greater risk of false negatives.

These results emphasize the critical role of primer selection in PCR-based surveillance and diagnosis of *T. lewisi*. The superior sensitivity of the LEW1S/LEW1R primers, particularly in detecting low-parasitemia infe-ctions without non-specific amplification, positions them as the most diagnostically robust option for field and laboratory use. The CATLew F/CATLew R set offers a valuable secondary option, while the TC121/TC122 set, originally designed for *T. cruzi*, is less suitable for species-specific applications due to occasional cross-reactivity.

The practical implications of this work are signi-ficant. Accurate detection of *T. lewisi* is essential for zoonotic risk assessment, early outbreak detection, and guiding public health responses, especially in urban and peri-urban environments like Banyuwangi, where rodent populations and poor sanitation intersect to increase spillover risk. The findings also have relevance for laboratory screening of rat colonies and regional One Health initiatives.

Among the strengths of this study are its field relevance, standardized laboratory protocols, and side-by-side performance metrics that provide clear guidance for molecular epidemiologists and diagnostic laboratories. The use of well-characterized primer sets under uniform conditions enhances the reproducibility and applicability of the results across Southeast Asia and beyond.

However, the study has limitations. The DNA samples were derived from archived specimens, and parasitemia levels were not quantified before PCR, which may have influenced detection rates. In addition, while microscopy served as a reference, its lower sensi-tivity may have misclassified some true infections, affe-cting calculated specificity. The analysis was limited to three primers; newer or modified primers could offer further improvements.

Future studies should aim to validate these findings across different geographic regions, rodent species, and ecological settings. Evaluating these primers in mixed species infections, conducting quantitative PCR to assess parasite load, and testing performance in human or non-rodent reservoirs would deepen understanding of *T. lewisi* transmission dynamics.

In conclusion, this study provides clear evidence that LEW1S/LEW1R is the most sensitive and specific primer set for the molecular detection of *T. lewisi* in wild rats. Its application in surveillance programs can enhance diagnostic accuracy, reduce false negatives, and improve early warning systems for zoonotic trypanosomosis. The findings contribute to bridging a critical diagnostic gap and support the broader implementation of molecular tools in public health surveillance and rodent-borne disease control.

## AUTHORS’ CONTRIBUTIONS

AY, AHW, GAIPS, and DHS: Conceptualization. AHW, FLP, AY, MAK, and AGRT: Sampling. AHW, FLP, AY, DHS, RE, RNP, and GAIPS: Sample analyses. AHW, FLP, AY, RE, DHS, GAIPS, MD, MAK, AGRT, and MM: Data analyses. AHW, GAIPS, FLP, DHS, AY, MD, and MM: Writing—original draft preparation. AHW, FLP, AY, DHS, RE, RNP, MD, and MM: Writing—review and editing. AHW, MD, and MM: Supervision. All authors have read and approved the final manuscript.
